# Construction of a Sensitive and Speed Invariant Gearbox Fault Diagnosis Model Using an Incorporated Utilizing Adaptive Noise Control and a Stacked Sparse Autoencoder-Based Deep Neural Network

**DOI:** 10.3390/s21010018

**Published:** 2020-12-22

**Authors:** Cong Dai Nguyen, Alexander E. Prosvirin, Cheol Hong Kim, Jong-Myon Kim

**Affiliations:** 1 Department of Electrical, Electronics and Computer Engineering (BK21Four), University of Ulsan, Ulsan 44610, Korea; dainc@mail.ulsan.ac.kr (C.D.N.); alpros91@mail.ulsan.ac.kr (A.E.P.); 2 School of Computer Science and Engineering, Soongsil University, Seoul 06978, Korea; cheolhong@ssu.ac.kr

**Keywords:** adaptive noise reducer, Gaussian reference signal, gearbox fault diagnosis, stacked sparse autoencoder–based deep neural network, varying rotational speed

## Abstract

Gearbox fault diagnosis based on the analysis of vibration signals has been a major research topic for a few decades due to the advantages of vibration characteristics. Such characteristics are used for early fault detection to guarantee the enhanced safety of complex systems and their cost-effective operation. There exist many fault diagnosis models that have been developed for classifying various fault types in gearboxes. However, the classification results of the conventional fault classification models degrade when they are applied to gearbox systems with multi-level tooth cut gear (MTCG) faults operating under variable shaft speeds. These conditions cause difficulty in discriminating the gear fault types. Due to the improved computational capabilities of modern systems, the application of deep neural networks (DNNs) is getting popular in a variety of research fields, such as image and natural language processing. DNNs are capable of improving the classification results even when addressing complex problems such as diagnosing gearbox MTCG faults. In this research, an adaptive noise control (ANC) and a stacked sparse autoencoder–based deep neural network (SSA-DNN) are used to construct a sensitive fault diagnosis model that can diagnose a gearbox system with MTCG fault types under varying shaft rotation speeds, despite its complicatedness. An ANC is applied to gear vibration characteristics to remove a significant level of noise along the frequency spectrum of vibration signals to fix the most fault-informative components of each fault case. Next, the autoencoder learns the gear faults characteristic features from these fault-informative components to separate the fault types considered in this study. Furthermore, the implementation of the SSA-DNN is substituted for feature extraction, feature selection, and the classification processes in traditional fault diagnosis schemes by high-performance unity. The experimental results show that the proposed model outperforms conventional methodologies with higher classification accuracy.

## 1. Introduction

Different types of gearboxes are used in various equipment such as vehicles, industrial machinery, and electrical generators. However, they are prone to defects due to harsh and continuous working conditions. Gear defects can lead to damage of the gearbox system and become a root cause of damaging the whole mechanical device, which may lead to serious economic losses and the threat of personal safety. Hence, the condition monitoring of gearboxes is essential, and it would be beneficial if the gear defects in gearboxes can be detected in the early stages. The general non-destructive method for condition monitoring of gearboxes is based on sensing the vibration characteristics which contain the fault-related components [1]. The complex sideband frequencies are distributed around the meshing frequency and its harmonics, which are considered as intrinsic components in the vibration signals and are used as informative components to identify gear defects [2,3]. From the standpoint of signal processing, a gearbox vibration signal is an amplitude and phase-modulated signal that occurs as many frequency tones centered by carrier frequencies are lined up along the whole range of the frequency spectrum. Each set of frequency tones contains a center frequency, as a meshing frequency or its harmonics, and sideband frequencies that are a function of the gear frequencies or specific oscillation frequencies distributed around the center frequencies. For diagnosing gearbox systems, it is essential to decompose the intrinsic fault-related components, and signal analysis is the most popular technique for these purposes. For capturing the vibration characteristics, accelerometers for measuring vibration signals are more frequently employed than acoustic emission sensors due to their relatively easy installation [4,5]. Notwithstanding, the vibration signals collected under variable rotational speeds in the gearbox are non-linear and non-stationary signals [6] which accommodate noise caused by the interaction of multiple related systems such as the resonance of shafts, gears, and other mechanical components, electrical and electronic control systems, data collection systems, and the environment [7]. These noise components are random and cause deterioration of the fault-relative characteristics in vibration signals, especially for the vibration signals of MTCG gearboxes (i.e., the noise frequency components might appear randomly with random amplitudes in the whole range of the frequency spectrum of a vibration signal and can cover or deform the original meshing frequency components, its harmonics, and sideband frequencies which are considered as fault signatures). For that reason, the appropriately selected signal processing techniques for reducing noise components and filtering out the informative components are of a high importance. 

Recently, many digital signal processing techniques have been developed by researchers that can be applied in different domains (e.g., time domain, frequency domain, and time-frequency domain) by employing a variety of advanced approaches such as Fourier transforms, short-time Fourier transforms, Hilbert transforms, wavelet transforms, Hilbert-Huang transform-based empirical mode decomposition [8,9,10,11,12,13,14], and the combined techniques [15,16,17]. The key methods which were utilized in those methodologies for discovering the fault-related components in the vibration signals are as follows: window filtering, thresholding, wavelet excitation, and intrinsic mode function extraction. These methods demonstrated their ability to reduce the noise at some ratio; however, the fault-informative components have been distorted as well. Due to these issues, these methods might not perform well in processing the signals containing MTCG faults to prepare the differentiable data for fault classification. Hence, in this paper, the ANC is utilized for processing the vibration signals to reduce the noise presence and preserve the fault-related components [18] to overcome the disadvantages of the previously introduced signal analysis models.

Considering the feature engineering and classification processes, the traditional gearbox fault diagnosis methods include feature pool configuration (feature extraction and feature selection) and fault classification by machine learning algorithms such as k-nearest neighbors (k-NN), support vector machines (SVMs), and artificial intelligence networks (ANNs) [19,20,21,22]. The main idea of those methods is to perform fault classification using the features which are statistical parameters extracted and selected from vibration signals in the time and frequency domains [23]. Feature extraction is an interfering process that requires a series of experiments for discovering fault-related discriminating feature parameters and then, based on their discriminating capabilities, the appropriated feature selection algorithms are applied for reducing the dimensionality of the constructed feature pool and selecting the most discriminative features for the classification process. These feature pool configuration processes can precede the difficultness of analyzing the vibration signals in each fault case of an MTCG gearbox system for extracting discriminative parameters. Moreover, these approaches can efficiently classify gear faults of a gearbox system under invariant shaft speed, but their performance degrades when applied to the datasets collected under varying shaft speeds. These issues can be addressed by creating a network that can efficiently determine tiny different components of non-stationary vibration signals of an MTCG in a gearbox system operating under varying speeds. The deep learning technique has dawned as an advantageous tool that has been applied in the fields of natural language processing, computer vision, image processing, and pattern recognition, and has succeeded in discriminating barely distinguishable components in categories through multiple non-linear transformations [24,25,26]. In other words, deep neural networks (DNNs) are suitable for use in the construction of sensitive and non-linear models. Instead of manually extracting the features and selecting the most separable ones, DNNs can be efficiently used for unsupervised hierarchical feature extraction and feature learning [27]. Thus, this study employs a stacked sparse autoencoder (SSA)-based DNN for identifying the fault types of an MTCG gearbox system based on the vibration signals with reduced noise components delivered by the ANC module.

The major contributions of this study are summarized as follows: (1) an adaptive noise control approach is designed for de-noising and preserving fault-related elements of raw vibration signals to obtain the optimized subbands on its outputs which mostly contain the essential informative components of vibration signals, and (2) the SSA-DNN utilizes the optimized subbands for identifying the MTCG defect types. The efficiency of the proposed model is evaluated by applying it to the vibration dataset collected from the MTCG gearbox that contains signals collected under six levels of tooth cut fault, such as 6.6%, 10%, 20%, 30%, 40%, and 50% cut as well as signals collected under normal operating conditions. The experimental dataset was collected under variable shaft rotating speeds, such as 300 RPM, 600 RPM, 900 RPM, and 1200 RPM, respectively. The results demonstrate the improved fault classification performance in comparison with the existing models. 

The rest of this paper is organized as follows. Section 2 presents a gearbox experimental dataset along with the characteristics of vibration for normal and defective gears. The detail of the proposed method is provided in Section 3. Section 4 describes the experiment configuration and the process of parameter tuning for the proposed network. Section 5 presents the results and discussion, and Section 6 contains the concluding remarks. 

## 2. The MTCG Gearbox Dataset

### 2.1. The Experimental Testbed and MTCG Gearbox Dataset

**Figure 1:**
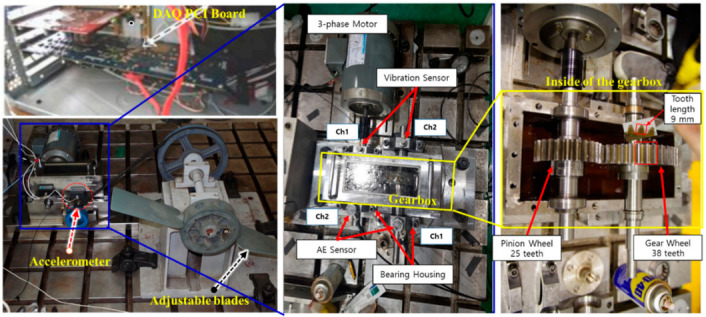
Experimental testbed arrangement for acquiring the MTCG gearbox dataset.

**Figure 2:**
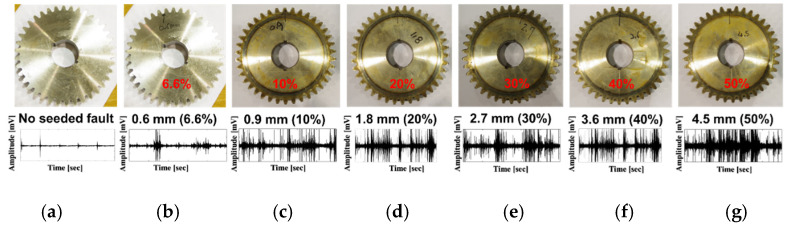
The defect states of the gear wheel and examples of vibration signals at a rotation speed of 600 RPM: (**a**) no seeded fault, normal gear, (**b**) tooth cut 6.6% (0.6 mm), (**c**) tooth cut 10% (0.9 mm), (**d**) tooth cut 20% (1.8 mm), (**e**) tooth cut 30% (2.7 mm), (**f**) tooth cut 40% (3.6 mm), and (**g**) tooth cut 50% (4.5 mm), respectively.

Figure 1 shows the experimental setup used for exploring the vibration characteristics of the MTCG gearbox system. A three-phase AC induction motor is connected to a pinion wheel through a drive shaft (DS) and a set of adjustable blades is mounted on a non-drive shaft (NDS) the other end of which is connected to a gear wheel. The numbers of teeth on the pinion wheel and the gear wheel are equal to 25 ($N_{p}$ = 25) and 38 ($N_{g}$ = 38), respectively. The length of each tooth is equal to 9 mm. The torque generated by the AC motor is transferred to the adjustable blade through the gearbox with a gear ratio of 25:38 (1:1.52). The multi-level tooth cut faults were seeded in one tooth of the gear wheel by cutting the percentage of the tooth length as depicted in Figure 2. The MTCG fault types contain a normal gear or a no seeded fault gear (N) condition, a tooth cut seeded gear defect of 6.6% (D1), a tooth cut seeded gear defect of 10% (D2), a tooth cut seeded gear defect of 20% (D3), a tooth cut seeded gear defect of 30% (D4), a tooth cut seeded gear defect of 40% (D5), and a tooth cut seeded gear defect of 50% (D6), respectively. For measuring the vibration characteristics of an MTCG gearbox in the normal and defects cases, the vibration sensor (an accelerometer 622B01 of IMI Sensor company) was installed at the end of the NDS, 72.5 mm from a gear wheel. Therewith, the shaft rotation speeds are monitored by using a displacement transducer (a speed sensor) to track the seeded hole in the DS once per rotation. The output signal from a vibration sensor was digitized using a PCI-based data acquisition board with a sampling frequency of 65,536 Hz continuously for one second. The data collection process was repeated 200 times to receive 200 samples of 1-s length per each gear defect state (seven states) under each shaft rotation speed. Therefore, the total number of observing samples is 5600, each of one second duration. The detailed description of the MTCG gearbox dataset is in Table 1. 

### 2.2. The Vibration Characteristics of the Gearbox System

**Figure 3:**
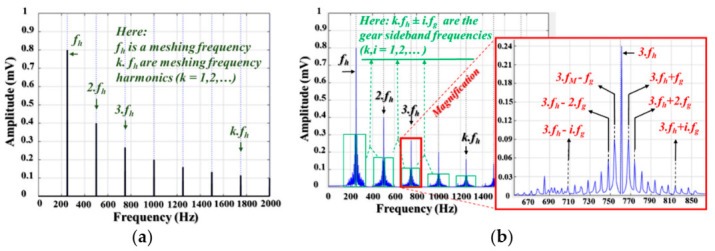
The example vibration signals present in the frequency domain: (**a**) a normal gearbox and (**b**) a defective gearbox.

The categories of gear defects can be generally split into three types: manufacturing defects (wheel eccentricity, defect of tooth profile, etc.), installation defects (parallelism), and defects caused by long-term operation (cracked tooth, spalled tooth, case ware tooth, tooth wear, etc.). In this work, the MTCG defects were created to simulate the operated defects as the multi-level depth of a tooth cut seeded in the gear wheel of the gearbox system. The vibration characteristics of a gearbox system are analyzed in the cases of a healthy gear (a defect-free gear) and a defect gear for identifying the informative fault-related components in the vibration signal. The vibration signal of a defect-free gear represents a linear and periodical signal that is calculated using the following formula [28]:

(1) $$y_{n}\left(t\right)\;=\overset{K}{\underset{k=i}{\sum }}Y_{k}\cos\left(2\pi kf_{h}t+\partial_{k}\right)\;$$

where $y_{n}\left(t\right)$ is a vibration signal of a healthy gear; K is a total number of meshing frequency harmonics in the observed frequency spectrum of a vibration signal; $Y_{k}$ and $\partial_{k}$ are the amplitude and phase of the *k*-th meshing frequency harmonics (*k* = 1,…, K); and $f_{h}$ stands for the meshing frequency which can be calculated using the parameters of a gear wheel ($f_{h}=f_{g}N_{g}$, where $f_{g}$ is a gear wheel rotation speed and $\;N_{g}$ is the number of gear teeth) or parameters of a pinion wheel ($f_{h}=f_{p}N_{p}$, where $f_{p}$ is a pinion wheel rotation speed and $N_{p}$ is the number of pinion teeth). Figure 3a illustrates an example of a frequency spectrum denoting the informative components as meshing frequency tones in a spectrum of vibration signals of a defect-free gearbox. 

Compared to a vibration signal of a normal gear, a signal of a defected gear is more complex due to the occurrence of impulsive vibrations when the motion is transferred from the DS to the NDS by rotating a pinion wheel through a gear wheel at a defective tooth position during one rotation cycle. Those periodical impulsive vibrations create the non-linear and non-stationary vibration signal formed as the amplitude and phase modulation signal in the point of view in the signal processing zone [3]. The fault gear vibration signal can be formulated [29] by Equation (2), and an example for demonstrating the fault-related informative components is shown in Figure 3b:

(2) $$y_{d}\left(t\right)\;=\overset{K}{\underset{k=0}{\sum }}S_{k}\left(1+\sigma_{k}\left(t\right)\right)\cos\left(2\pi kf_{h}t+{∊}_{k}+\psi_{k}\left(t\right)\right)$$

Here, $\sigma_{k}\left(t\right)\;=\overset{N}{\underset{i=0}{\sum }}\Theta_{ki}\cos\left(2\pi if_{g}t+\Omega_{kj}\right)$ and $\psi_{k}\left(t\right)=\overset{N}{\underset{i=0}{\sum }}\Psi_{ki}\cos\left(2\pi if_{g}t+\xi_{ki}\right)$ are modulating components of the amplitude and phase partial in the fault gear vibration signal $y_{d}\left(t\right)$; $\Theta_{ki}$,$\Psi_{ki}$ are amplitudes and $\Omega_{kj}$,$\xi_{ki}$ are phases of the *i*-th sideband, respectively, roundly k-order meshing the frequency tone of the vibration signal $y_{d}\left(t\right)$. 

## 3. The Incorporated Construction Model of the ANC and the SSA-DNN

**Figure 4:**
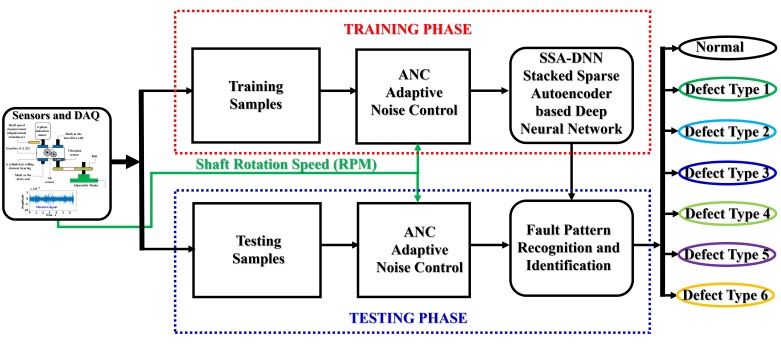
A block diagram of the proposed gearbox fault diagnosis model.

The proposed sensitive and speed invariant model for diagnosing gearbox faults is presented in Figure 4. Three major function blocks are utilized in this model, such as the data collection system (Sensors and DAQ), the ANC, and the SSA-DNN. The data collection system collects the vibration dataset of an MTCG gearbox system for each fault type (seven fault types in total) under variable shaft rotation speeds. It collects the vibration data samples and captures the gear defect behaviors in the vibration characteristics: each vibration sample is evenly acquired during one second to monitor several complete rotation cycles of the defected gear. The ANC module then processes the raw vibration signals. Firstly, it performs down-sampling three times along with filtering the signal with a low-pass filter to receive the vibration subbands within the frequency range from 0 to 10 kHz according to the real operating frequency range of the vibration sensor [18]. The expression of multi-level gear defect types on the vibration characteristic is signified by the magnitudes of the principal frequency tones, therefore the main function of the ANC is optimizing vibration subbands for removing the redundant components along with noise while preserving the original fault-related components. The output of the ANC provides the optimized subband in the frequency domain (power spectrum density) which mostly contains the meshing frequency, its harmonics, and their distributed sideband gear frequency tones (i.e., the defect-related informative components). Under variant speeds condition, the positions of principal frequency tones are altered according to the explanation in Section 2. There exist the components that represent the speed invariant MTCG defects as the numbers of latent features related to the ratio and proportional to the amplitudes and displacements in the optimized vibration subbands, which are difficult to extract features from by traditional methodologies [30]. Notwithstanding, based on the unsupervised learning and hierarchy of feature extraction constitution of a deep neural architecture (DNA), the SSA-DNN can vanquish the issue and automatically explore the most defect-substantial features from a set of components in the frequency spectrums of optimized subbands output from the ANC. By fetching out these features, the SSA-DNN can use them to identify defect types of an MTCG gearbox system for achieving a high classification result in the output layer. 

### 3.1. Adaptive Noise Control (ANC)

**Figure 5:**
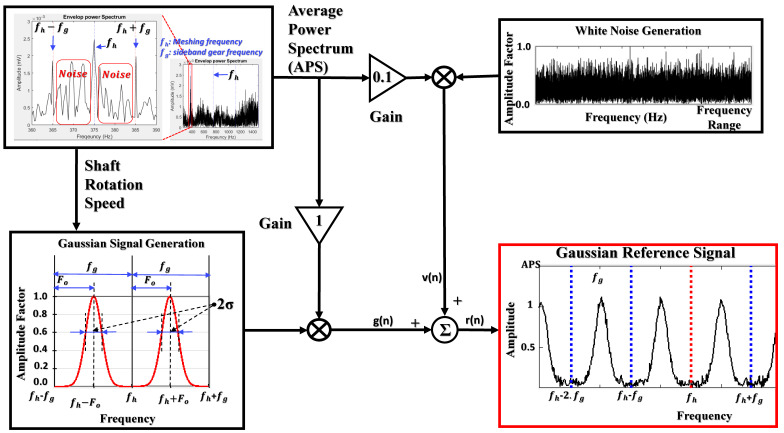
A functional block scheme of generating the adjustable Gaussian reference signal.

ANC is a signal processing method used for reducing noise and preserving the fault-related informative elements in gearbox vibration characteristics. The ANC approach is a self-constructed and time-varying system that uses a recursive algorithm for optimizing its parameters for obtaining the desired optimized signal in its output [31]. General ANC consists of a digital filter, an adaptive algorithm, and a reference signal generator. An adaptive algorithm operates to update the coefficients of the digital filter based on the feedback error signal of a filtered reference and an input signal to receive the optimized denoised subband signal in the output of the ANC [32]. In this study, the ANC employs the adaptive noise reducer-based Gaussian reference signal (ANR-GRS) which has been elaborated in [18] for reducing noise and optimizing gearbox vibration signals. An adaptive noise control scheme contains two inputs (the desired input and a reference input) and one output. As the desired input for the observed signal, the vibration subband is used in this study, while the reference input is used for a signal that imitates the parasitic noise in the observed signal. The function of the ANC approach can be described in detail in the following processes [18]:

1. Generating the reference signal to supply to the reference input of an ANC:

Mainly, there are two types of noise present in the vibration signal: white noise and band noise. Hence, the reference signal generator creates the output signal behavior which is homologous with those such as Gaussian signals and white noise signals, as illustrated in Figure 5. The parameters of a Gaussian signal (a mean and a standard deviation value) can be adjusted based on the input variable of the shaft rotation speed. The adjustable Gaussian window, a component for building the entire Gaussian signal, is drawn to adapt to the frequency space between two consecutive sideband gear frequencies, formulated as follows:

(3) $$W_{\mathrm{Gref}}\left(p\right)\;=\overset{N_{t}}{\underset{p=1}{\sum }}e^{-\frac{{\left(p-\;F_{o}\right)}^{2}}{2\sigma^{2}}}$$

where the adjustable parameters (mean value $F_{o}$ and standard deviation value σ) are functions of the shaft rotation frequency [18]. Concretely, $F_{o}$ is proportional to the frequency of faulty wheel ($f_{DG}$) and can be computed as below:

(4) $$F_{o}\;=\;\varepsilon \cdot f_{DG},$$

and by linearizing the Gaussian function, the standard deviation is approximated to the mean value as:

(5) $$\sigma =0.318\cdot F_{o}=0.318\cdot \varepsilon \cdot f_{DG}.$$

Also, the number of sideband segments $N_{t}$ is calculated using the known parameters such as the number of samples $N_{s}$, sampling frequency $F_{s}$, and fault wheel frequency. The formulation of sideband segments is presented below:

(6) $$N_{t}\;=\frac{2N_{s}}{F_{s}}\cdot f_{DG}$$

where the frequency of a faulty wheel ($f_{DG}$) is represented as a gear frequency ($f_{g}$) which is defined in Section 2. Therefore, by adjusting the ratio coefficient  ε, the Gaussian window can access the space between two consecutive sideband frequencies in the frequency spectrum of a vibration signal to reduce the presence of noise. According to specific conditions defined in [18], first, the coefficient ε is selected from the range of [0.25 0.75], and then, the Gaussian windows are created with the parameters chosen as shown below:

(1) the mean value $F_{o}$ is assigned to be in the range:
  (7) $$0.25\cdot f_{DG}\leq \;F_{o}\;\leq 0.75\cdot f_{DG}$$
(2) the standard deviation of the Gaussian windows is selected in the following range:
  (8) $$\sigma =\left\{\begin{array}{c} 0.318\cdot \varepsilon \cdot f_{DG}\;\;\;\;\;\;\;\;\;\;\;\;\;\;\;\;\;\;\;\;\;\;when\;0.25\leq \varepsilon \leq 0.5\\ 0.318\cdot (1-\varepsilon )\cdot f_{DG}\;\;\;\;\;\;\;\;\;\;\;\;\;\;\;\;\;when\;0.5<\varepsilon \leq 0.75 \end{array}\right.$$

By limiting the adjusting values of the coefficient  ε, each generated Gaussian window is positioned completely inside the area between two consecutive sideband frequencies during the optimization processes in the next steps. This ensures that the adaptive noise control technique performs reducing band-noise significantly whereas originally preserving the fault-related informative components as meshing frequencies, its harmonics, and sideband frequencies [18].

2. The construction of an adaptive filter

**Figure 6:**
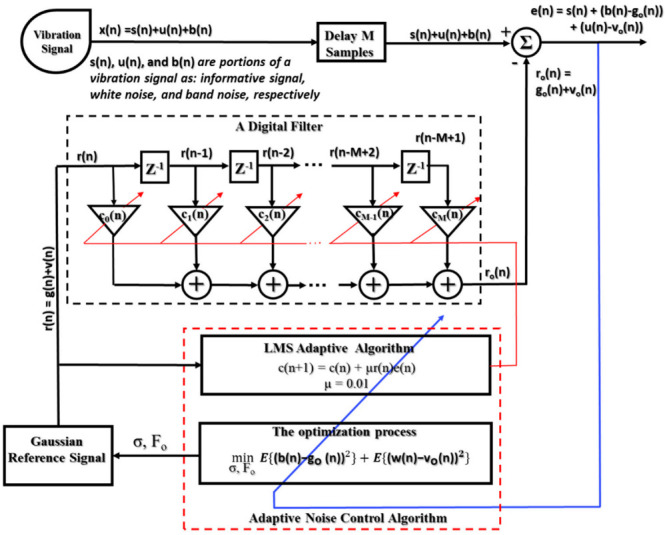
A functional block diagram of an adaptive noise control module.

The adaptive filter is formed by combining the N-tap FIR digital filter (the coefficient vector as c(n) ≡ [c_0_, c_1_, …, c_N-1_]^T^) and a least mean square (LMS) adaptive algorithm. The reference signals are used as the input to the digital filter and its output signals are summed with the vibration subbands to calculate the output error signals. Based on this error, the LMS adaptive algorithm tunes the coefficient vectors according to the convergence criterion of the least mean square error for determining the optimal coefficient vector (c_0_) and then identifying the local optimal subbands. The operation of an adaptive filter is functionally described in Figure 6.

3. The optimization process for selecting the optimal vibration subband

Each vibration subband, processed by an adaptive filter with the input reference of a parameter- adjustable Gaussian reference signal, results in many subbands in its output (termed as local optimal subbands) corresponding to the set of specific values of parameters and appropriate optimal coefficient vectors. At this step, the ANC selects the subband which has a minimum mean squared value as an output result of the optimization process (termed as an optimized subband) illustrated in Figure 6. This optimized output subband is a final output of the ANC module that contains mostly the fault-related informative components and trivial disturbances or redundant components.

In fact, the signal portions, which reflect the gear states (a meshing frequency, meshing frequency harmonics, and gear sideband frequencies), are represented mostly in the frequency domain as magnitudes, tones amplitudes, oscillations, frequencies, and the ratios between them. Thus, it is suitable to use the frequency spectrum of the optimized subband as the input data to the SSA-DNN so the deep network can explore and automatically extract the defect characteristic features from its inputs. Additionally, the usage of the frequency spectrum of the vibration signal reduces the complexity of the DNN. Therefore, in this paper, the frequency spectrum of the optimized vibration subband calculated by Fourier transform [33] is used as the input of the SSA-DNN module. The spectrum of the optimized subband is of ranges from 0 to 10 kHz due to the down-sampling process of raw one second vibration samples.

### 3.2. Stacked Autoencoder 

**Figure 7:**
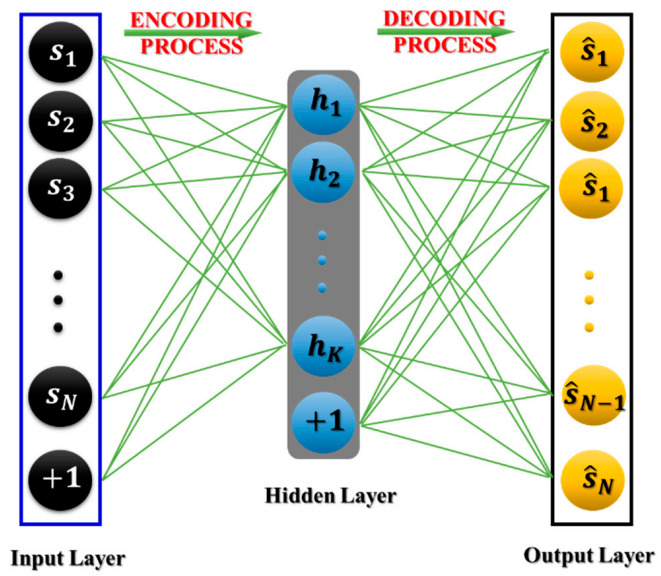
The diagram of the two learning processes of an autoencoder.

A stacked autoencoder is a type of DNN, with a number of hidden layers greater than one, formed by stacking simple autoencoders for feature discrimination and classification. To understand the concept of a stacked autoencoder, a simple autoencoder should be discussed first. It is an unsupervised DNN based on a three-layer symmetrical architecture for learning the representation of high-level data [34]. An autoencoder functions through two learning stages-encoding and decoding, as shown in Figure 7. In the encoding stage, it transforms the higher-dimensional input into a lower-dimensional one. High-dimensional input data is compressed by the hidden layer in DNN architecture [35]. Hence, the encoding path contributes to the principal goal of an autoencoder. In the mathematical expression, the higher-dimensional input represented as $s\in R^{N}$ (i.e., *N* dimensions) is encoded to a lower-dimensional space $h\in R^{K}$ (i.e., *K* dimensions), producing the output vector known as a latent space. The encoder function or the latent space can be represented as follows:

(9) $$h=f_{e}\left(W_{e}s+b_{e}\right),$$

where $\;f_{e}$, $W_{e}$, and $b_{e}$ are the encoding activation function, weights, and bias of the network, respectively. From Figure 7, it can be interpreted that the decoding portion reconstructs the output of a lower-dimensional space that was compressed from higher-dimensional input using an encoding process. The reconstruction procedure can be expressed as follows:

(10) $$\hat{s}=f_{d}\left(W_{d}h+b_{d}\right)$$

Here $f_{d}$, $W_{d},$ and $b_{d}$ are the decoding activation function, weights, and bias of the network, respectively. The key goal of the autoencoder is to minimize the reconstruction loss which is an objective function of an autoencoder. It can be expressed as following [36]:

(11) $$L\left(s,\hat{s}\right)\;=\pm \left(\left||s-\hat{s}|\right|\right)\;=\;||s-f_{d}\left(W_{d}\left(f_{e}\left(W_{e}s+b_{e}\right)\right)+b_{d}\right)||$$

In this paper, the feature engineering and classification path of the sensitive and speed invariant gearbox fault diagnosis model is constructed by stacking multiple sparse autoencoders as a stacked sparse autoencoder (SSA) for determining the small differences of features between gear defect types which are the basis components for improving classification accuracy. In the next subsection, the sparse autoencoder algorithm is explained.

### 3.3. Sparse Autoencoder

Sparsity is a special parameter of autoencoders, which puts a constraint onto the hidden layer and causes activation of inactive hidden units to discover the tiny differences in decimated features of data representation more sensitively and robustly than the simple autoencoder architecture [37]. The constraint of a sparse autoencoder usually embeds a regularization term to the objective function. Therefore, the regularized objective function can be expressed as follows [36]:

(12) $$L\left(s,\hat{s}\right)\;=\frac{1}{N}\overset{N}{\underset{n=1}{\sum }}\overset{K}{\underset{k=1}{\sum }}\left(s_{kn}-\hat{s}\right)+\beta \times \phi_{weights}+\;\gamma \times \Phi_{sparse}$$

In Equation (12), β and γ refer to the $L_{2}$ regularization coefficient and the sparsity penalty factor, respectively. In the training process of an autoencoder, it is sometimes observed that the value of γ alters in an inversed way with the values of weight parameters and behaves proportionally to the latent space h (for example the value of the sparsity penalty factor increases by decreasing the value of weights and increasing the value of latent code). Thus, the $L_{2}$ regularization is introduced for embedding in the cost function to solve this issue, which can be represented as follows [36]:

(13) $$\phi_{weights}=\frac{1}{2}\overset{L}{\underset{l}{\sum }}\overset{n}{\underset{i}{\sum }}\overset{k}{\underset{j}{\sum }}{\left(W_{ij}^{l}\right)}^{2}$$

where L, n, and k represent the number of hidden layers, the number of observations, and the number of variables in the input data, respectively. Consequently, the sparsity constraint $\Phi_{sparse}$ can be formulated as follows:

(14) $$\Phi_{sparse}=\overset{L^{\left(1\right)}}{\underset{i=1}{\sum }}KL(\rho ||\bar{\rho })=\overset{L^{\left(1\right)}}{\underset{i=1}{\sum }}(\rho \log\frac{\rho }{\rho_{i}}+\left(1-\rho \right)\log(\frac{1-\rho }{1-\rho_{i}}))$$

where

(15) $$\rho_{i}=\frac{1}{m}\overset{m}{\underset{j=1}{\sum }}z_{i}^{1}\left(s_{j}\right)\;=\frac{1}{m}\overset{m}{\underset{j=1}{\sum }}h(w_{i}^{\left(1\right)T}s_{j}+b_{i}^{\left(1\right)})$$

This Equation (14) is known as Kullback-Leibler divergence [38]. $\Phi_{sparse}$ takes a higher value when the i-th neuron gives an average activation value $\bar{\rho }$ because that deviates mainly from the desired value ρ.

**Figure 8:**
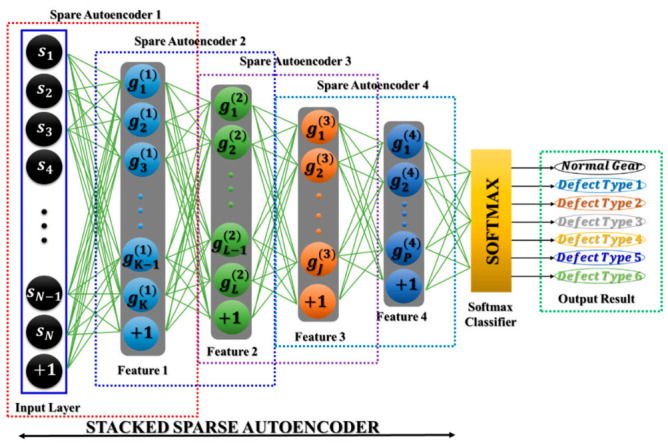
The DNA of a Stacked Sparse Autoencoder.

To establish the SSA, several numbers of sparse autoencoders, which have been individually trained, are stacked and positioned in a form such the input layer is placed before the series of hidden layers, and a SoftMax classifier [39] represents an output layer of this network architecture. Hence, all sparse autoencoders, which are stacked, form the DNA. Figure 8 depicts an example of a DNA with four hidden layers for visual understanding. This DNA first operates in an unsupervised learning manner, where all of the SSAs extract useful features and then, in a supervised learning manner, the DNA executes fine-tuning employing a back-propagation algorithm based on the stochastic gradient descent [40]. After the training process is completed, the unseen data is used for evaluating the performance of the DNA.

## 4. Experimental Setup and Tuning DNA Parameters

To validate the effectiveness of feature engineering and classification by the SSA-DNN in the proposed model, we perform a set of four experiments listed in Table 2. In these experiments, the SSA-DNN uses the input data as the samples of the frequency spectrum of the subbands that were optimized by the ANC. The four subsets of gearbox data were taken based on shaft rotation speed, i.e., each data subset contains 1400 samples in total for all defect states (200 samples for each class of seven defect states: N, D1, …, D6), which were acquired from the vibration sensor when the shaft rotates at the same speed. For each experiment trial, the proposed DNA was trained numerous times with diverse numbers of epochs using samples corresponding to one speed of the shaft and validated with the dataset collected under two other shaft speeds, then changing samples belonging to different speeds for all four experiments.

### 4.1. Tuning Parameters for the SSA-DNN

The parameters of the DNA play an important role in classification performance, so that the tuning process for selecting the optimal values has to be performed [41]. To construct this model, we have repeatedly tested the proposed model using various values of model parameters such as the length of recipient input, the sparsity regularization term, the number of hidden layers, the number of hidden nodes, and the cost function to evaluate their effect on DNA performance. The following subsections explain the parameter tuning process in detail. 

#### 4.1.1. Exploration of the DNA Parameter Configurations

The length of the recipient input is the size of a single sample which is inputted to the DNA, it is also known as the value of higher-dimensional representation of the input layer. According to [41], this parameter is the first important factor for recognizing the complex features that can be well supported for the classification of MTCG fault types to build up the sensitive gearbox fault diagnosis model. Therefore, a larger recipient input length helps the DNA to extract better representative features. Nevertheless, a huge size of the input increases the computational complexity of the model, while a reasonable size of the input can provide both a reasonable quality of feature extraction and well-proportioned computation complexity. As mentioned in Section 3.1, the one second raw vibration signals were sampled at a frequency of 65,536 Hz, resulting in 65,536 points in the time domain. This raw signal was preprocessed by three-time down sampling accompanied by low-pass filtering before entering the ANC module. Hence, there are 21,845 (65,536/3) data points in the optimized time-domain signals received in the output of the ANC module. By applying the Fourier transform to these signals, the symmetrical frequency spectrum of each optimized subband containing an imaginary part (this part represents a spectrum of the signal in the negative frequency) and a real part (for the frequency tones greater than zero) is received. The real part that represents a real frequency spectrum of an optimized subband with 10,922 (21,845/2) data points is used as the input to the DNA. The usage of a large number of data points at the input layer might increase the computational complexity; however, the effectiveness of fault identification might not be improved significantly. On the contrary, a further reduction of the input size will lead to the reduction of frequency resolution and hence, it might cause challenges for the model when identifying the MTCG defect types. Thus, the length of the recipient input with 10,922 points of an optimized subband represents a rational trade-off between the classification performance and computational complexity for the sensitive and speed invariant MTCG gearbox fault diagnosis model. 

**Figure 9:**
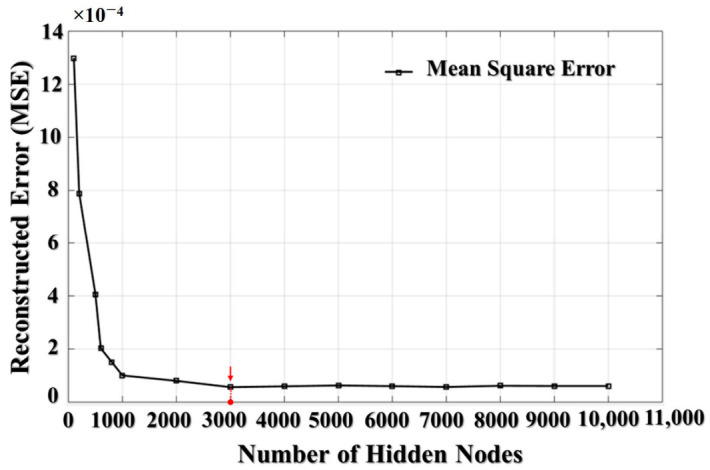
The dependence graph of reconstructed error MSE and the number of nodes in the first hidden layer.

Similarly, the number of neurons in the hidden layers also influences the performance of the DNA. Although there are no exact guidelines for selecting the number of neurons for a hidden layer of an autoencoder, this parameter directly impacts the process of feature extraction. Based on the functionality of the autoencoder, the number of nodes in the first hidden layer has to be lesser than the length of the recipient input for compressing the higher-dimensional data. To adjust the parameters of node number and sparsity, in this paper we create a fine-tuning dataset which is formed by randomly picking 100 data instances corresponding to each class under each rotation speed condition. Hence, the fine-tuning dataset consisted of $N_{samp}\times N_{class}\times N_{speed}=100\times 7\times 4=2800$ data instances in total. Figure 9 illustrates the relationship between the reconstruction error curve and the number of nodes for the first hidden layer obtained while training the autoencoder on the fine-tuning dataset during 350 epochs. This curve demonstrates that the number of 3000 nodes in the hidden layer, which is greater than 20% of the input size (10,922), leads to smaller reconstruction errors. A further increase in this number minorly affects the reconstruction error, but the computational complexity would be increased significantly. Thus, it is recommended to keep the number of nodes for the hidden layer at less than 35% of the input size. This criterion is applied to the remaining hidden layers in the proposed model, so the number of nodes in each consecutive hidden layer is in the range from 20% to 35% of the number of nodes in the previous layer.

**Figure 10:**
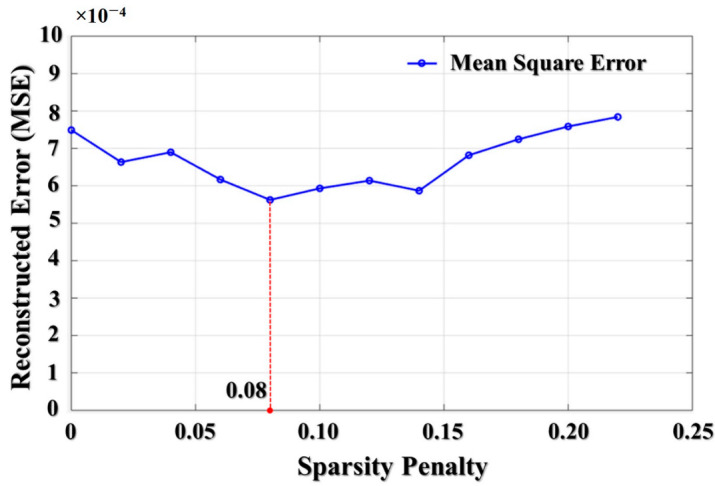
The relation graph between the sparsity term and the reconstruction MSE.

The sparsity penalty can be used for improving the forward learning process of an unsupervised autoencoder, whose purposive activity orients to manifest the highly representative features. To evaluate the effect of the sparsity penalty, the reconstruction error is mostly considered for the experiment the value of sparsity penalty parameter in the first autoencoder (the first hidden layer is selected with number hidden nodes as 3000). Figure 10 demonstrates the relation between the value of the sparsity term and the reconstruction error, which is a mean square error (MSE) in this study, achieved when training the autoencoder on a fine-tuning dataset during 350 epochs. It is observed that values of sparsity penalty in the range from 0.05 to 0.15 are better than the remaining values, and a value of 0.08 is the optimal one leading to the minimum MSE. Hence, this value has been chosen as a penalty factor for all the hidden layers in the proposed model. 

The number of hidden layers plays an important role in the learning process. There exists a general opinion that a higher number of hidden layers results in better accuracy, but also reduces the generalization ability of the network [40]. In this work, a series of experiments to determine the number of hidden layers were performed while varying their number from three to six, as shown in Table 3. From this table, it can be observed that a number of hidden layers greater than three leads to the smallest reconstruction errors. Regarding a higher number of hidden layers, the reconstruction error does not change significantly; however, the computational time can be increased dramatically when making the architecture deeper. Therefore, to select a suitable number of hidden layers, the time performance also should be considered. 

**Figure 11:**
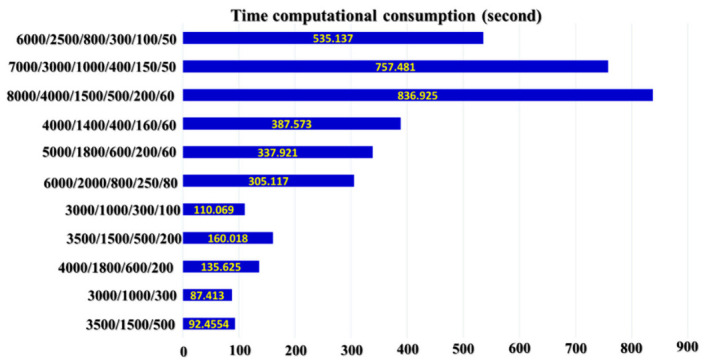
The training time consumption of SSA-DNN architectures with different numbers of hidden layers and nodes in them.

The complexity of computation of the architecture, in general, can be measured as an average time required for one training cycle of DNA. Figure 11 shows the time consumption of different SSA-DNN deep architectures with various numbers of hidden layers and nodes in them during the training process. In this figure, the DNAs with higher numbers of hidden layers and nodes requires more time for training due to the depth of the architecture.

#### 4.1.2. Parameter Selections of the SSA-DNN Model

**Figure 12:**
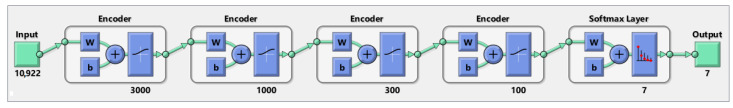
The final architecture of the SSA-DNN model.

Through the experiments in the previous subsection, it was observed that with the increase of DNA architecture complexity, the reconstruction error was getting smaller while the time needed for training the deep architecture was increasing. However, from Table 3 it can be seen that after reaching certain numbers of hidden layers and nodes, the further increase of architecture complexity leads only to minor reductions of the reconstruction error. From this observation, it can be concluded that the actual number of highly representative features is limited, and thus, when the DNA attempts to extract more features from its input, which might be redundant and not representative, they would not affect the resulting reconstruction error significantly. The structure of a DNA should contain several numbers of hidden layers to adequately perform dimensionality reduction of the input data, where each hidden layer analyzes its input to perform both feature extraction and selection to receive the higher-level representative features. These features are then used for discriminating the MTCG defect types during the classification process. Because of the challenge of constructing the speed invariant fault diagnosis model for MTCG gearbox systems, the parameters are selected to prioritize the small reconstruction error with acceptable execution time consumption. Regarding the architectures with five or six hidden layers, the reconstruction errors are relatively small in comparison with other architectures, though, the time consumed for the training process is much higher and the error values are not much larger. Therefore, in this study, the number of hidden layers is selected as four with the amounts of nodes (i.e., number of features) in them as 3000, 1000, 300, and 100 neurons for the first, second, third, and fourth hidden layers, respectively. The finalized optimal parameters of the SSA-DNN model are listed in Table 4, and its architecture is shown in Figure 12.

## 5. Result and Discussion

**Figure 13:**
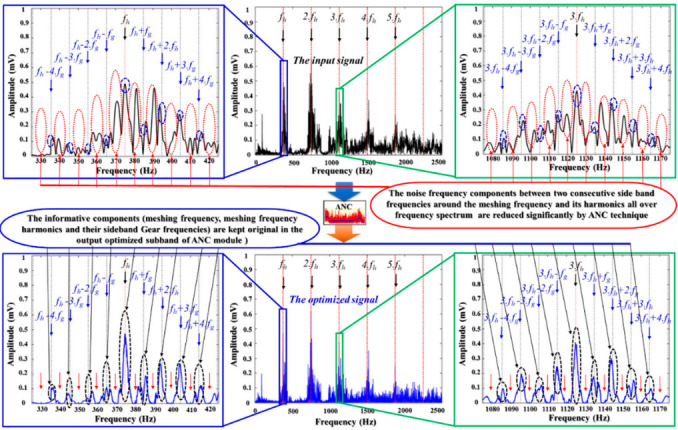
Frequency spectrum analysis of the vibration subband (for fault state D2 at 900 RPM) in the comparison between an input and output subband of the ANC module.

The main function of the ANC is to perform noise reduction and to preserve the fault-related useful components existing in the vibration signals. To collect the informative content of the vibration sample, where the content represents numerous fault-related components that are useful for designing the sensitive fault diagnosis model, the analog signals from the vibration acceleration sensor were digitized with a high sampling frequency of 65,536 Hz every one second. Thus, a 1-sec length data sample is used to monitor several rotation cycles (from three to thirteen rotational cycles depending on the rotation speed from 300 RPM to 1200 RPM) to collect fault-related vibration characteristics with some special oscillations. After data collection, the digitized vibration samples were filtered by a digital low-pass filter with the cut-off frequency of 10,000 Hz accompanied with the down-sampling process to remove the high-frequency components (i.e., components located in spectrum higher than 10,000 Hz) which are out of operation range of the acceleration sensor, and to preserve the vibration components with intrinsic fault-informative features following realistic operation of a gearbox system. That is the first step for preprocessing data to remove the redundancy in the raw vibration signals. The vibration subbands output from a low-pass filter are inputted into the ANC module for a fine-optimizing process for noise reduction. In the range of the frequency spectrum less than 10 kHz, the ANC uses adaptive windows to access and remove white noise and band noise remaining between two consecutive sideband frequencies along the frequency spectrum. Figure 13 demonstrates the superiority of the ANC module for the de-noising process. Here, the red dotted circles indicate the noise frequency component zones of the input signals which were reduced significantly in the optimized subband outputted from the ANC. Moreover, the amplitudes of the sideband frequency tones, the meshing frequency, and its harmonics are kept unchanged when the vibration subband flows through the ANC module (the dashed blue and black circles). The outputs of the ANC are the optimized vibration subbands represented in the frequency domain for the expression of the energy distribution. These spectra are used as inputs to the SSA-DNN module for extracting the representative latent features by an unsupervised learning technique, the autoencoder, which is a part of the SSA-DNN module.

**Figure 14:**
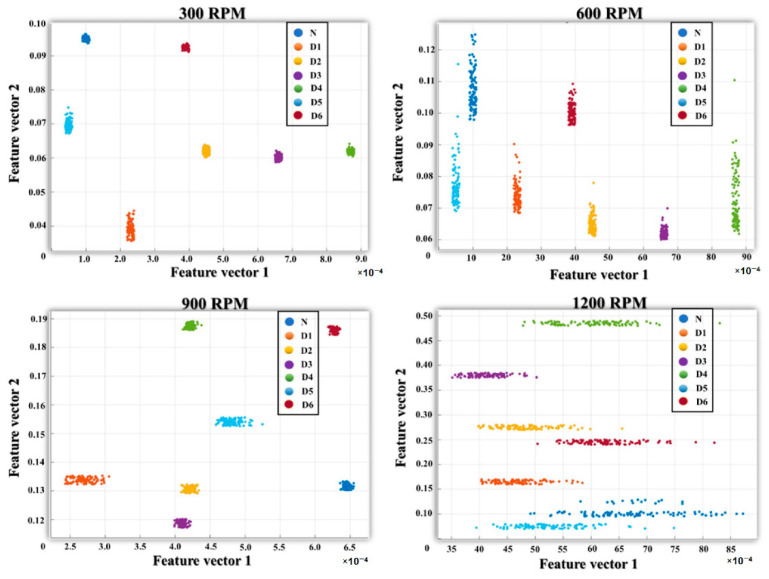
The feature space distributions of seven defect types using the features extracted by sparse autoencoders under four various rotational speeds.

Figure 14 illustrates feature spaces for seven defect types of an MTCG gearbox using some of the discriminative features extracted by sparse autoencoders from the frequency spectra of optimized subbands under different rotational speeds. This figure shows that the data instances corresponding to different signal classes are well separable in feature space. Here, the samples belonging to one defect type are placed closely, whereas the samples of different defect types are located separately in the visualized feature space. These distinct features are extracted by stacking the sparse autoencoder layers and are used to enhance the performance of the deep architecture using a back-propagation algorithm to minimize the reconstruction errors and then, finally, to classify gearbox defects. For fault diagnosis performance evaluation, we compared the results of the proposed model with previous models such as ANC and SVM [18] (model 1), ANC and ANN (model 2), stacked denoising autoencoder [42] (model 3), and the spectra imaging of vibration signal [43] (model 4).

These results are presented in Table 5. The performance is evaluated using the four cases of experiment setup expressed in Table 2. The training dataset of each experiment contains 1400 vibration samples (200 vibration samples for each defect state of seven states as N, D1, D2, D3, D4, D5, D6) for each rotational speed to construct the deep architecture network model. The testing process is performed by 2800 vibration samples of two different rotational speeds. By executing four experiments, the vibration samples of four rotational speeds are used for training set in sequence, whereas two datasets of rotational speeds, which are different from rotational speed in training dataset in each experiment, are consumed for the testing process. In these experiments, models 1 and 2 use the statistical features extracted from time and frequency domains, whereas the remaining models use autonomous feature extraction methods based on the unsupervised learning approach (model 3) and vibration imaging approach (model 4). Models 1 and 2 use the optimized subband output from an ANC module to extract twenty-one feature parameters and then, using these feature vectors, classify fault types using SVM and ANN, respectively. Manually extracted features in models 1 and 2 cause a challenge when classifying multi-level tooth cut gear defects. Their fault classification results were around 68% ± 10% for model 1 and 59.4% ± 10% for model 2, fluctuating over four experiments. The construction of DNA in model 3 is performed by replacing the four sparsity autoencoder hidden layers with two layers of denoising autoencoders using the optimal regularization terms and parameters from [42] and removing the ANC module from the proposed model. In model 3, the input data are the vibration subbands outputted from the down sampling and low-pass filtering process, with the denoising and feature engineering processes performed using the objective functions with the embedded manifold regularization. The fault identification results achieved by this model were about 82.88% ± 8% in four experiments.

These results can be observed because many fault-related components stay hidden in the background noise which can only be detected by the application of signal processing methods. Regarding model 4, the raw 1-sec vibration signal with 65,536 points was firstly down-sampled by four times with a 10 kHz low-pass filter integrated for antialiasing to obtain the vibration subband with 16,384 data points. Then this subband is segmented in series without overlap by using windows of 1024-point size to attain sixteen segments of 1024-point vibration subbands. Then, each 1024-point window containing the vibration subband is transformed from the time domain to the frequency domain by FFT to obtain a 513-point sized vibration frequency spectrum. This process was repeated eight times by randomly picking eight segments of 1024-point vibration subbands from sixteen segments. These spectrums were stacked to form the 513 × 8 grayscale image corresponding to each raw 1-sec vibration sample. This image was later converted to a binary image by an 8 × 4 sized filter and the threshold (0.7). Hence, the binary image containing 4014 components in the frequency domain was used as the input to the ANN with three layers (input, hidden with three nodes, and output layers) for classification. The fault classification results of model 4 on the dataset used in this paper were about 45.17% ± 6% during four experiments. By analyzing the experimental results of the referenced models, it can be seen that the sensitive and speed invariant fault diagnosis model proposed in this study outperformed their fault diagnosis performance with results around 97% ± 2% during four experiments showing small accuracy deviations when alternating the shaft rotational speeds of the MTCG gearbox system.

Additionally, to verify the stability of the proposed algorithm, the experiments described above have been performed five times. The classification accuracies and their averages computed over five experimental trials are presented in Table 6. From these results, it can be seen that the proposed model demonstrates stable fault classification accuracy in independent trials of the experiments performed for training and testing subsets containing samples collected under different operating conditions, i.e., rotating speed.

Controlling the noise embedded in the vibration signals is essential for the sensitive detection of multi-level cut tooth faults in gearbox systems. The presence of a high noise level can cause misidentifications of fault types and thus reduce the fault classification accuracy. Noise reduction is a complex problem, and it is not always possible to completely resolve this issue by signal processing or feature engineering techniques. Therefore, simultaneous usage of the ANC and SSA-DNN methods is an efficient approach for significant noise reduction while preserving the original fault-related information of the gear vibration characteristic, which is useful for fault identification. The design of a sensitive and speed invariant model requires exploration of the representative features that can be used for discrimination of multi-level tooth cut gear defects and maintaining its reliable performance under the operating speed fluctuation conditions in the gearbox system. In general, the manual feature extraction methods cannot satisfy those requirements, thus the unsupervised approaches based on deep neural networks are well-suitable for extracting the latent representative features by the process of minimizing reconstruction errors during the operation of a back-propagation algorithm in the DNA. The SSA-based DNN constructed in this research satisfies the requirements for constructing the proposed model, such as extracting the representative feature space, selecting the most defect-related useful features for classification, and finally, achieving high fault classification results.

## 6. Conclusions

This study presents a novel method which combines an ANC and an SSA-DNN to utilize their advantages for constructing a sensitive and speed invariant fault identification model for gearbox systems with multi-level tooth cut gear defects. The ANC technique is created based on the analysis of vibration characteristics of a gearbox system to generate the speed-dependent reference window signals with adjustable parameters, according to the noise types presenting in the raw vibration signals. Then, these generated window series were adaptively adjusted to access the space between two consecutive defect-related frequency tones and remove the noise along the whole frequency range of vibration signals. The ANC optimizes the input vibration signal for outputting the optimal subband which contains mostly the defect-related frequency tones with the integration of low-level background noise. Then, the frequency spectra of these optimal subbands are used as the input to the deep network architecture. This network is built up by stacking sparse autoencoders as the hidden layers of the network and using a Softmax activation function at the output layer for extracting latent representative feature spaces and selecting the most defect-related discriminative features for identifying the multi-level tooth cut fault types under the condition of various shaft rotational speeds. The effectiveness of the proposed model is validated by experiments performed using the vibration dataset containing MTCG gearbox defects collected under four different rotational speeds. To validate the property of speed invariance for the proposed model, the experiment was arranged as four sub-experiments using the datasets corresponding to each rotational speed. Each sub-experiment uses a one-speed dataset to construct and train the model. Then this given model is used for fault identification using two datasets collected under other speed conditions. This procedure was performed four times using the different speed datasets for building the model in each. The average classification result achieved over four experiments was 97%, which outperforms the techniques used for comparison. Moreover, the classification results shown by the proposed model did not fluctuate significantly (2–3%) when applied to different speed datasets, which evidences that the prosed model is speed invariant and can be used for identifying multi-level tooth cut defects in a gearbox system under varying rotational speeds.

## Figures and Tables

**Table 1:** A detailed description of the MTCG defect types and dataset.

Gearbox Fault State.	Description	Number of 1-S Data Samples Acquired for Each Rotation Speed				Sampling Frequency (Hz)
		300 RPM	600 RPM	900 RPM	1200 RPM	
Normal Gear (N)	No seeded fault in the teeth of a gearbox	200	200	200	200	65,536
Defect type 1(D1)	Gear tooth cut 6.6% (0.6 mm)	200	200	200	200	65,536
Defect type 2(D2)	Gear tooth cut 10% (0.9 mm)	200	200	200	200	65,536
Defect type 3(D3)	Gear tooth cut 20% (1.8 mm)	200	200	200	200	65,536
Defect type 4(D4)	Gear tooth cut 30% (2.7 mm)	200	200	200	200	65,536
Defect type 5(D5)	Gear tooth cut 40% (3.6 mm)	200	200	200	200	65,536
Defect type 6(D6)	Gear tooth cut 50% (4.5 mm)	200	200	200	200	65,536

**Table 2:** Description of the dataset for training and testing with RPM in the experiment setup.

The Experiments	Number of Samples	The RPM of Data Samples
Experiment 1	Training sample: 1400	The shaft speed: 300 RPM
	Testing samples: 2800	The shaft speed: 600 RPM and 900 RPM
Experiment 2	Training sample: 1400	The shaft speed: 600 RPM
	Testing samples: 2800	The shaft speed: 900 RPM and 1200 RPM
Experiment 3	Training sample: 1400	The shaft speed: 900 RPM
	Testing samples: 2800	The shaft speed: 300 RPM and 1200 RPM
Experiment 4	Training sample: 1400	The shaft speed: 1200 RPM
	Testing samples: 2800	The shaft speed: 300 RPM and 600 RPM

**Table 3:** The reconstruction error with the sets of numbers of hidden layers and their nodes.

Number of Hidden Layers	Nodes per Each Layer	Reconstruction Error
3	3500/1500/500	16.312 × 10^−3^
3	3000/1000/300	15.189 × 10^−3^
4	4000/1800/600/200	9.745 × 10^−5^
4	3500/1500/500/200	6.887 × 10^−5^
4	3000/1000/300/100	4.698 × 10^−5^
5	6000/2000/800/250/80	3.783 × 10^−5^
5	5000/1800/600/200/60	4.2 × 10^−5^
5	4000/1400/400/160/60	4.034 × 10^−5^
6	8000/4000/1500/500/200/60	1.439 × 10^−5^
6	7000/3000/1000/400/150/50	1.907 × 10^−5^
6	6000/2500/800/300/100/50	2.543 × 10^−5^

**Table 4:** The optimal selected parameters for constructing the SSA-DNN model.

Input Size (Sample Length)	Number of Layers	Number of Nodes	Sparsity Constraint	Activation Function
10,922	4	3000, 1000, 300, 100	0.08, 0.08, 0.08, 0.08	Logistic sigmoid

**Table 5:** Classification results of the referenced and proposed models in four experiments based on various rotating speed data.

Models	Training Set (1400 Samples)	Test Set (2800 Samples)	Accuracy
**I**	300 RPM	600 RPM, 900 RPM	62.78
	600 RPM	900 RPM, 1200 RPM	79.83
	900 RPM	300 RPM, 1200 RPM	67.13
	1200 RPM	300 RPM, 600 RPM	62
	Average accuracy by four experiments		**68**
**II**	300 RPM	600 RPM, 900 RPM	65
	600 RPM	900 RPM, 1200 RPM	48.10
	900 RPM	300 RPM, 1200 RPM	73.5
	1200 RPM	300 RPM, 600 RPM	51
	Average accuracy by four experiments		**59.4**
**III**	300 RPM	600 RPM, 900 RPM	90.66
	600 RPM	900 RPM, 1200 RPM	79
	900 RPM	300 RPM, 1200 RPM	85.50
	1200 RPM	300 RPM, 600 RPM	76.35
	Average accuracy by four experiments		**82.88**
**IV**	300 RPM	600 RPM, 900 RPM	41.15
	600 RPM	900 RPM, 1200 RPM	39.55
	900 RPM	300 RPM, 1200 RPM	48.26
	1200 RPM	300 RPM, 600 RPM	51.72
	Average accuracy by four experiments		**45.17**
**The proposed model**	300 RPM	600 RPM, 900 RPM	95.51
	600 RPM	900 RPM, 1200 RPM	97.32
	900 RPM	300 RPM, 1200 RPM	99
	1200 RPM	300 RPM, 600 RPM	96.1
	Average accuracy by four experiments		**97**

**Table 6:** Fault classification results of the proposed model obtained during five experimental trials.

Training Set (1400 Samples)	Testing Set (2800 Samples)	Experiment Trials					Average Accuracy (%)
		#1	#2	#3	#4	#5	
300 RPM	600 RPM 900 RPM	93	96.87	95.7	93.85	98.15	95.51
600 RPM	900 RPM 1200 RPM	97.68	98.2	94.95	100	95.78	97.32
900 RPM	300 RPM 1200 RPM	100	100	99.68	98.19	97.15	99.00
1200 RPM	300 RPM 600 RPM	98.00	94.28	95.47	97.9	94.87	96.10

## Data Availability

Data available on request due to restrictions. The data presented in this study are available on request from the corresponding author. The data are not publicly available.

## References

[1] McNames J. (2002). Fourier Series Analysis of Epicyclic Gearbox Vibration. J. Vib. Acoust..

[2] Baxter J.W., Bumby J.R. (1995). An Explanation for the Asymmetry of the Modulation Sidebands about the Tooth Meshing Frequency in Epicyclic Gear Vibration. Proc. Inst. Mech. Eng. Part C J. Mech. Eng. Sci..

[3] Chaari F., Bartelmus W., Zimroz R., Fakhfakh T., Haddar M. (2012). Gearbox Vibration Signal Amplitude and Frequency Modulation. Shock Vib..

[4] Mitchell J.S. (1991). An Introduction to Machinery Analysis and Monitoring. Comput. Eng..

[5] Ghodake S.B., Mishra A.K., Deokar A.V. (2016). A Review on Fault Diagnosis of Gear-Box by Using Vibration Analysis Method. IPASJ Int. J. Mech. Eng..

[6] Bartelmus W., Zimroz R. (2009). A New Feature for Monitoring the Condition of Gearboxes in Non-Stationary Operating Conditions. Mech. Syst. Signal Process..

[7] Patil C.R., Kulkarni P.P., Sarode N.N., Shinde K.U. (2017). Gearbox Noise & Vibration Prediction and Control. Int. Res. J. Eng. Technol..

[8] Randall R.B. (1987). Frequency Analysis.

[9] Randall R.B., Antoni J., Chobsaard S. (2001). The Relationship between Spectral Correlation and Envelope Analysis in the Diagnostics of Bearing Faults and Other Cyclostationary Machine Signals. Mech. Syst. Signal Process..

[10] Aharamuthu K., Ayyasamy E.P. (2013). Application of Discrete Wavelet Transform and Zhao-Atlas-Marks Transforms in Non Stationary Gear Fault Diagnosis. J. Mech. Sci. Technol..

[11] Kang M., Kim J., Kim J.M., Tan A.C.C., Kim E.Y., Choi B.K. (2015). Reliable Fault Diagnosis for Low-Speed Bearings Using Individually Trained Support Vector Machines with Kernel Discriminative Feature Analysis. IEEE Trans. Power Electron..

[12] Loutridis S.J. (2004). Damage Detection in Gear Systems Using Empirical Mode Decomposition. Eng. Struct..

[13] Zhang C., Peng Z., Chen S., Li Z., Wang J. (2018). A Gearbox Fault Diagnosis Method Based on Frequency-Modulated Empirical Mode Decomposition and Support Vector Machine. Proc. Inst. Mech. Eng. Part C J. Mech. Eng. Sci..

[14] Buzzoni M., Mucchi E., D’Elia G., Dalpiaz G. (2017). Diagnosis of Localized Faults in Multistage Gearboxes: A Vibrational Approach by Means of Automatic EMD-Based Algorithm. Shock Vib..

[15] Liu B., Riemenschneider S., Xu Y. (2006). Gearbox Fault Diagnosis Using Empirical Mode Decomposition and Hilbert Spectrum. Mech. Syst. Signal Process..

[16] Goharrizi A.Y., Sepehri N. (2012). Internal Leakage Detection in Hydraulic Actuators Using Empirical Mode Decomposition and Hilbert Spectrum. IEEE Trans. Instrum. Meas..

[17] Han G.D., Wan S.T., Lv Z.J., Liu R.H., Wang J., Tang G.J. (2014). The Analysis of Gearbox Fault Diagnosis Research Based on the EMD and Hilbert Envelope Demodulation. Adv. Mater. Res..

[18] Nguyen C.D., Prosvirin A., Kim J.M. (2020). A Reliable Fault Diagnosis Method for a Gearbox System with Varying Rotational Speeds. Sensors.

[19] Lei Y., Zuo M.J. (2009). Gear Crack Level Identification Based on Weighted K Nearest Neighbor Classification Algorithm. Mech. Syst. Signal Process..

[20] Han D., Zhao N., Shi P. (2019). Gear Fault Feature Extraction and Diagnosis Method Under Different Load Excitation Based on EMD, PSO-SVM and Fractal Box Dimension. J. Mech. Sci. Technol..

[21] Samanta B. (2004). Gear Fault Detection Using Artificial Neural Networks and Support Vector Machines with Genetic Algorithms. Mech. Syst. Signal Process..

[22] Ajanalkar S.S., Bute P.V., Shrigandhi G.D. (2017). Gear Fault Prediction by Using Artificial Neural Network (ANN)—A Review. https://inpressco.com/wp-content/uploads/2017/05/Paper1355-58.pdf.

[23] Rauber T.W., De Assis Boldt F., Varejão F.M. (2015). Heterogeneous Feature Models and Feature Selection Applied to Bearing Fault Diagnosis. IEEE Trans. Ind. Electron..

[24] Afaq Ali Shah S., Bennamoun M., Boussaid F. (2016). Iterative Deep Learning for Image Set Based Face and Object Recognition. Neurocomputing.

[25] Hinton G.E., Salakhutdinov R.R. (2006). Reducing the Dimensionality of Data with Neural Networks. https://science.sciencemag.org/content/313/5786/504.

[26] Sarikaya R., Hinton G.E., Deoras A. (2014). Application of Deep Belief Networks for Natural Language Understanding. IEEE Trans. Audio Speech Lang. Process..

[27] Zhang R., Peng Z., Wu L., Yao B., Guan Y. (2017). Fault Diagnosis from Raw Sensor Data Using Deep Neural Networks Considering Temporal Coherence. Sensors.

[28] Fan X., Zuo M.J. (2006). Gearbox Fault Detection Using Hilbert and Wavelet Packet Transform. Mech. Syst. Signal Process..

[29] Fakhfakh T., Chaari F., Haddar M. (2005). Numerical and Experimental Analysis of a Gear System with Teeth Defects. Int. J. Adv. Manuf. Technol..

[30] Caesarendra W., Tjahjowidodo T. (2017). A Review of Feature Extraction Methods in Vibration-Based Condition Monitoring and Its Application for Degradation Trend Estimation of Low-Speed Slew Bearing. Machines.

[31] Widrow B., Stearns S.D. (1985). Adaptive Signal Processing.

[32] Lee K.A., Gan W.S., Kuo S.M. (2009). Subband Adaptive Filtering: Theory and Implementation.

[33] Bracewell R.N. (1986). The Fourier Transform & Its Applications.

[34] Saufi S.R., Ahmad Z.A.B., Leong M.S., Lim M.H. (2019). Low-Speed Bearing Fault Diagnosis Based on ArSSAE Model Using Acoustic Emission and Vibration Signals. IEEE Access.

[35] Bengio Y., Lamblin P., Popovici D., Larochelle H. (2007). Greedy Layer-Wise Training of Deep Networks. Adv. Neural Inf. Process. Syst..

[36] Sohaib M., Kim J.M. (2018). Reliable Fault Diagnosis of Rotary Machine Bearings Using a Stacked Sparse Autoencoder-Based Deep Neural Network. Shock Vib..

[37] Saufi S.R., Ahmad Z.A.B., Leong M.S., Lim M.H. (2020). Gearbox Fault Diagnosis Using a Deep Learning Model with Limited Data Sample. IEEE Trans. Ind. Inf..

[38] Van Erven T., Harremoës P. (2014). Rényi Divergence and Kullback—Leibler Divergence. IEEE Trans. Inf. Theory.

[39] Zhao W., Wang Z., Lu C., Ma J., Li L. Fault Diagnosis for Centrifugal Pumps Using Deep Learning and Softmax Regression. Proceedings of the 2016 12th World Congress on Intelligent Control and Automation (WCICA).

[40] Aggarwal C.C. (2018). Neural Networks and Deep Learning.

[41] Coates A., Lee H., Ng A.Y. (2011). An Analysis of Single-Layer Networks in Unsupervised Feature Learning. J. Mach. Learn. Res..

[42] Yu J.B. (2019). Evolutionary Manifold Regularized Stacked Denoising Autoencoders for Gearbox Fault Diagnosis. Knowl. Based Syst..

[43] Amar M., Gondal I., Wilson C. (2015). Vibration Spectrum Imaging: A Novel Bearing Fault Classification Approach. IEEE Trans. Ind. Electron..

